# Methylation Status of the Adeno-Associated Virus Type 2 (AAV2)

**DOI:** 10.3390/v11010038

**Published:** 2019-01-09

**Authors:** Renáta Tóth, István Mészáros, Daniela Hüser, Barbara Forró, Szilvia Marton, Ferenc Olasz, Krisztián Bányai, Regine Heilbronn, Zoltán Zádori

**Affiliations:** 1 Institute for Veterinary Medical Research, Centre for Agricultural Research, Hungarian Academy of Sciences, Hungária krt. 21, H-1143 Budapest, Hungary; toth.renata13@gmail.com (R.T.); meszaros.istvan@agrar.mta.hu (I.M.); barbara.forro@gmail.com (B.F.); marton.szilvia@agrar.mta.hu (S.M.); olasz.ferenc@agrar.mta.hu (F.O.); banyai.krisztian@agrar.mta.hu (K.B.); 2 Hungarian National Blood Transfusion Service, Laboratory of Transplantation Immunogenetics, Karolina út 19-21, H-1113 Budapest, Hungary; 3 Institute of Virology, Campus Benjamin Franklin, Charité Medical School, Hindenburgdamm 27, 12203 Berlin, Germany; daniela.hueser@charite.de (D.H.); regine.heilbronn@charite.de (R.H.)

**Keywords:** AAV2, adeno-associated virus, bisulfite PCR, CpG methylation, DNA virus, Parvoviridae

## Abstract

To analyze the methylation status of wild-type adeno-associated virus type 2 (AAV2), bisulfite PCR sequencing (BPS) of the packaged viral genome and its integrated form was performed and 262 of the total 266 CG dinucleotides (CpG) were mapped. In virion-packaged DNA, the ratio of the methylated cytosines ranged between 0–1.7%. In contrast, the chromosomally integrated AAV2 genome was hypermethylated with an average of 76% methylation per CpG site. The methylation level showed local minimums around the four known AAV2 promoters. To study the effect of methylation on viral rescue and replication, the replication initiation capability of CpG methylated and non-CpG methylated AAV DNA was compared. The in vitro hypermethylation of the viral genome does not inhibit its rescue and replication from a plasmid transfected into cells. This insensitivity of the viral replicative machinery to methylation may permit the rescue of the integrated heavily methylated AAV genome from the host’s chromosomes.

The *Parvoviridae* family consists of small, single-stranded DNA viruses with 4–6 kb linear genomes. It is a very diverse virus family with the capability to infect a wide range of hosts from insects to mammals [1]. Adeno-associated dependoparvoviruses (AAVs) are separated from other parvoviruses by their CpG island-like genome structure with high GC content (>50%) and high observed/expected CpG ratio (>70%) [2]. AAVs are also distinguished from other parvoviruses by their different reproductive strategy, because they require the presence of an unrelated helper DNA virus for successful reproduction. In the absence of a helper virus, they can establish a latent infection by preferentially integrating into the open chromatin structures of the host’s genome or remaining latent as nuclear episomes [3,4].

AAVs are among the most frequently used gene therapy vectors, because they can infect many tissues in the human body without known adverse effects [5]. During the first months, recombinant AAV-mediated gene transfer results in a peak of transgene expression, but later this level decreases and reaches a reduced steady-state level [6,7]. Since CpG methylation can inhibit transcription [8], the methylation pattern of the promoter and vector in episomal adeno-associated dependoparvovirus A (AAV2)-based gene therapy constructs have been examined, but no significant CpG methylation has been found [9]. The methylation status of the replicative and the integrated form of the wild-type AAV2 remained unknown.

We previously determined that the genome of Ungulate protoparvovirus 1 (PPV) remains hypomethylated during the entire viral life cycle independent of its tissue of origin, and in vitro CpG methylation has no significant effect on viral replication [2]. The different reproductive strategy and the strikingly different genome composition of the AAV2 (AAV has 266 CpG sites, 54% GC content and 0.78 observed/expected CpG ratio (oCpGr) value compared to the 60 CpG sites, 38% GC content and 0.33 oCpGr of the PPV) suggested that CpG methylation may have a more significant role in the life cycle of the AAV2 than in the life cycle of the PPV. Therefore, we sought to investigate the methylation status of wild-type AAV2 genome during the different stages of the viral life cycle including the packaged viral DNA and the integrated and excisable form of the genome.

AAV2 virions were produced as previously described [10] by co-transfecting pTAV2-0 [11] and pDG [12] into HEK-293 cells. Freeze-thaw lysates were treated with benzonase (Merck, Darmstadt, Germany) to degrade non-encapsidated DNA, and AAV genomes were purified using proteinase K (Carl Roth, Karlsruhe, Germany) and phenol/chloroform extraction. The integrated viral genome was purified from latently infected Detroit 6 cells [13] using lysis buffer (1% N-lauroylsarcosine, 25 mM Tris-Cl pH 8.5, 10 mM EDTA pH 8.0) and proteinase K treatment followed by repeated phenol/chloroform extractions and ethanol precipitation.

To detect and separate the integrated form of the genome from spontaneously released AAV genomes, total Detroit 6 cell DNA was run on an agarose gel. Despite the typical low molecular weight AAV bands of 4.7 replicative form 1 (RF1) or 9.4 kb (RF2) were not being detected the high molecular weight chromosomal DNA was isolated by the Zymoclean Gel DNA Recovery Kit (Zymo Research, Irvine, CA, USA), as recommended by the manufacturer.

The methylation pattern of the AAV genomes derived from total Detroit 6 cell DNA, from the isolated high molecular weight DNA, and from the packaged viral DNA was determined by bisulfite PCR. The bisulfite treatment of the encapsidated, single-stranded DNA was performed with the EpiTect Bisulfite Kit (Qiagen, Venlo, The Netherlands) according to the manufacturer’s instructions. Treatment of the genomic DNA was optimized by adding an extra denaturation step (95 °C, 5 min) followed by incubation at 60 °C for 2 h. The conversion efficiency of the unmethylated cytosines was verified by Sanger sequencing of several PCR fragments from the 27 CpG sites containing fragment AAV11 (Table 1). Sanger sequencing was performed with the BigDye Terminator v3.1 Cycle Sequencing Kit (Applied Biosystems, Foster City, CA, USA), according to the manufacturer’s recommendations.

For the amplification of the modified CpG-containing DNA fragments, 22 PCR primer pairs were designed using the MethPrimer program [14] (Table 1). The 22 PCR fragments covered all CpGs of the AAV genome except the first and the last two sites (262 out of 266). DNA amplifications of most of the fragments were carried out by an initial denaturation for 5 min at 95 °C, followed by 35 cycles at 95 °C for 20 s, 52 °C for 20 s, and 72 °C for 20 s by using DreamTaq DNA Polymerase (Thermo Fisher Scientific, Waltham, MA, USA). For certain PCR fragments, the thermal conditions were altered. The temperature of the elongation step was changed to 58 °C at the 6th, 10th, 14th, 18th, 21st and 22nd fragments (Table 1), while the elongation occurred at 60 °C in the case of the 2nd and 20th fragments. The amplified fragments were purified from 1.2% agarose gel using the Zymoclean Gel DNA Recovery Kit. Finally, the PCR fragments were pooled in equal amounts and were sequenced with an Ion Torrent PGM sequencer. The CLC Genomics Workbench 7.0.4 was used for data analysis. The average read length was 213 nucleotides and 262 (of the total 266) CpG sites were mapped. The read depth of the 262 CpG sites of the virion-packaged DNA, the AAV genome from the total DNA and the AAV genome from the isolated chromosomal DNA were between 112 and 12603, 49 and 4335, and 71 and 4953, respectively.

In virion-packaged DNA, the ratio of the methylated cytosines was between 0–1.7% with an average of 0.6% methylation/CpG sites. In contrast, despite the CpG island-like genome structure, the integrated AAV2 genome was found to be hypermethylated, and the methylation ratio of the CpG sites varied between 20.4% and 98.3% with an average of 76% methylation per site (Figure 1a). Sequencing of the isolated high molecular weight DNA yielded very similar results: the methylation of the CpG cytosines was between 21% and 98.8% with an average of 78.2% methylation per site (Figure 1b). Minimal differences (0.003–12.3%) were detected in the methylation status of CpGs determined from total cellular DNA or isolated chromosomal DNA, confirming that the overwhelming majority of the detected methylation pattern derived from integrated copies and not from episomal forms.

The methylation level showed local minimums around the four promoters (p5, p19, p40 and p81) and the least methylated CpG sites were found in the X protein-coding ORF (Figure 1b,c). It is tempting to speculate that the lower level of methylation of these CpG sites might play a functional role in the reactivation of the promoters.

Our results indicate that the packaged and replicating AAV DNA is hypomethylated, as has been shown for other parvoviruses (PPV, B19) [2,18] and small- or medium-sized DNA viruses (e.g., papillomaviruses, adenoviruses) [19]. Hypomethylation is a characteristic feature of the replicating of small DNA viruses, despite the fact that unmethylated CpGs may provide an access of the host immune system to immunostimulatory, unmethylated CpGs during in vivo replication and cell lysis. It is likely that hypomethylation is the result of rapid replication, compartmentalization or active exclusion of the DNA methylases by the viral proteins from the replicating DNA [19].

Although the hypermethylation of the latently integrated AAV genome is not fully unexpected, it is somewhat surprising. Some of the earlier observations indeed implied methylation. Usually, newly integrated replication-incompetent viral fragments inserted into the host genome become rapidly methylated. Complete and replication-competent retrovirus sequences are also recognized by the host defense system (e.g., Daxx protein) and integrated proviruses are rapidly silenced by antiviral epigenetic responses including histone modification and DNA methylation [20].

On the other hand, the AAV2 genome was reported to integrate into transcriptionally active open chromatin regions and in CpG islands [4,21] and it can be released from latently infected Detroit 6 cells by helper virus infection [13]. Furthermore, the AAV genome has a CpG island-like genome composition that in the host genome most frequently remains unmethylated, and its methylation silences gene expression [22,23]. Thus, these data may suggest that the unique CpG island-like structure of the AAV genome evolved to avoid methylation and keep the open chromatin structure of the integrated genome to ensure easy access for transcription factors to viral promoters. However, our findings challenge this hypothesis.

For replication initiation, Rep proteins are needed to release the integrated AAV DNA from the host genome [24,25,26,27,28]. DNA hypermethylation is usually associated with transcriptional repression. Accordingly, the crucial question is how the RNAs of the viral Rep proteins are transcribed from the methylated integrated copies to supply the required proteins, especially because several methylation-sensitive transcription sites are localized in, or in close proximity of, the AAV promoters (Figure 1).

To further analyze how methylation influences viral rescue, we compared the replication initiation capability of CpG methylated and non-CpG methylated AAV DNA. The pTAV2-0 plasmid produced in bacteria supplied the non-CpG methylated genome (although it contained bacterial DAM and DCM methylation). For the production of CpG methylated AAV DNA, the pTAV2-0 plasmid was linearized by FastDigest *Eco*RV restriction enzyme (Thermo Fisher Scientific, Waltham, MA, USA) and in vitro methylated using the CpG methylase kit (Zymo Research, Irvine, CA, USA). The reaction mix included 2 µg DNA, 4 µL of 10× CpG Reaction Buffer, 6 µL of 20× SAM (12 mM), 2 µL of 4 U/µL CpG Methylase (M.SssI)) and distilled water to a final volume of 40 µL, and was incubated overnight at 30 °C.

The efficiency of hypermethylation was estimated to be more than 90% by the ImageJ program [29] after comparing the intensity of the linearized methylated undigested and the methylation-sensitive SsiI-enzyme digested (Thermo Fisher Scientific, Waltham, MA, USA), vector bands (Figure 2a, lanes 5 and 2 respectively).

Linearized methylated and unmethylated plasmids were transfected together with pHelper plasmid [30] in equal amounts (0.5 µg each) into HEK-293 cells by TurboFect reagent (Thermo Fisher Scientific, Waltham, MA, USA) in triplicate according to the supplier’s recommendations. Transfection of the unmethylated plasmid without pHelper was carried out as a negative control, also in triplicate. At 4, 24, 48 and 72 h post-transfection, the viral DNA was extracted from 200 µL tissue supernatant by the High Pure Viral Nucleic Acid Kit (Roche, Basel, Switzerland) according to the manufacturer’s recommendations. The titer of progeny viruses was compared by qPCR from three independent transfection experiments. The PCR conditions were the following: initial denaturation for 5 min at 95 °C, followed by 30 cycles at 95 °C for 20 s, 64 °C for 20 s, and 72 °C for 20 s using DreamTaq DNA Polymerase, EvaGreen (Biotium, Fremont, CA, USA) DNA binding dye and a primer set (forward: 5′-TGC GTA AAC TGG ACC AAT GAG AAC-3′; reverse: 5′-TGT TGG TGT TGG AGG TGA CGA TCA-3′). The Mann–Whitney U test was applied for the statistical analysis of the data.

The result indicates that in vitro CpG hypermethylation of the viral genome does not inhibit its rescue from a plasmid. It also minimizes the possibility that helper rescue of integrated AAVs could be the result of the activation of incidentally existing un-methylated episomes [31,32] in these cells rather than the rescue of the integrated methylated genome. Hypermethylation has even a biologically minor but statistically significant positive effect (Figure 2b) on the output virus titers at 48 h and 72 h (*p* = 0.00058 and *p* = 0.00018).

Recently, it was found that AAV2 latency is mediated by rapid heterochromatin formation by the heterochromatin hallmark trimethylated histone 3 lysine 9 (H3K9me3) and the chromatin regulating KAP1 protein [33]. In addition to H3K9me3, the CpG hypermethylation of the DNA is one of the most characteristic features of the heterochromatin [34]. Accordingly, our data—that the integrated AAV2 is hypermethylated in Detroit 6 cells—give additional support to the heterochromatinization of the latent AAV2 genome.

Despite being hypermethylated, AAV2 is rescuable from Detroit 6 cells. We demonstrated that AAV indeed can be rescued even from in vitro hypermethylated plasmid DNA. Yet, the question can be raised of whether the results obtained from “naked plasmids” can be extrapolated to the chromatinized AAV genome [35]. However, transfected plasmid DNA, just like the nonintegrated wild-type AAV genome, is rapidly associated with histones and chromatinized [36], which makes it highly probable that similar mechanisms permit the rescue of the heavily methylated integrated AAV genome from transfected plasmids or from the host’s chromosomes.

It is widely accepted that the binding of YY1 and MLTF to p5 is a key factor in the establishment and maintenance of latency [37,38]. However, the binding of these transcription factors to DNA is methylation-sensitive [39,40] and the effect of methylation to p5 binding was not considered in the original studies in the early 1990s. A recent publication of the epigenetic regulation of AAV latency [37] and our present data may warrant the reinvestigation of the role of these transcription factors in the maintenance of the latency of the methylated genome.

A voluminous literature demonstrates that the CpG methylation of the promoter regions is strongly associated with transcriptional repression, and DNA methylation is dominant over other epigenetic mechanisms for regulating gene expression. However, it is still unclear whether the changes in DNA methylation are the cause or the consequence of the altered gene expression [41,42]. Further studies of the methylated AAV genome release from latency can provide additional valuable data about the relationship between CpG methylation and the dynamics of the chromatin structure.

## Figures and Tables

**Figure 1:**
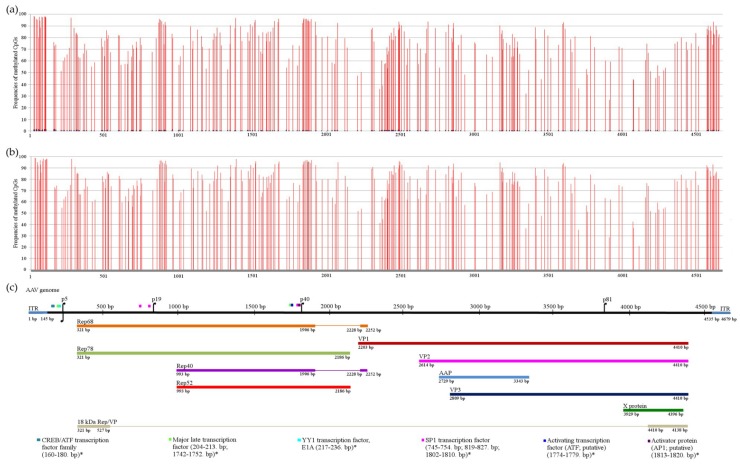
Deep sequencing of the bisulfite treated adeno-associated virus type 2 (AAV2) genomes. Vertical bars label the position of the CpGs in the AAV2 genomes in the diagrams: (**a**) Methylation values of the packaged AAV2 genome and the integrated AAV2 genome from total Detroit 6 DNA are represented by blue and red bars, respectively; (**b**) methylation values of the integrated AAV2 genome from purified chromosomal DNA; (**c**) the AAV2 genome and its transcription–translation map [15,16,17] is presented in scale showing the CpG-containing binding sites of transcription factors as well. The methylation-sensitive transcription factors are labelled by an asterisk.

**Figure 2:**
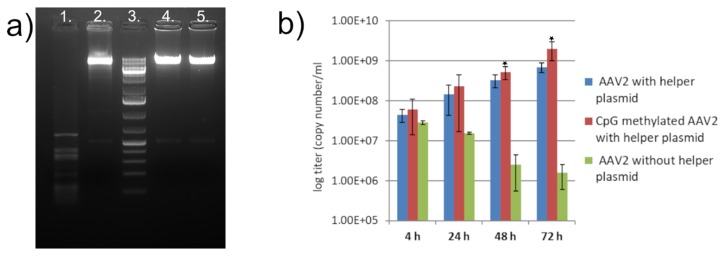
Replication initiated by differently methylated AAV DNAs: (**a**) digestion of the differently methylated pTAV2-0 DNAs. Lane 1, CpG unmethylated *Eco*RV linearized vector digested with SsiI (cutting at 57 sites); lane 2, CpG methylated *Eco*RV linearized vector digested with SsiI; lane 3, GeneRuler 1 kb Plus DNA Ladder; lane 4, CpG unmethylated *Eco*RV linearized vector; lane 5, CpG methylated *Eco*RV linearized vector; (**b**) copy numbers of the viral genome in the supernatant of cells transfected by differently methylated AAV2 plasmids. Vertical bars indicate twice the standard deviation in each case.

**Table 1:** Primers used for bisulfite PCR.

Primer Name	Sequence	Product Size (bp)	CpGs in Product
AAV1F	5′-TTGGTTATTTTTTTTTTGCGCGTT-3′	205	19
AAV1R	5′-CCTCTAATACAAAACCTCCCTA-3′		
AAV2F	5′-GGGTTAGGGAGGTTTTGTATT-3′	279	17
AAV2R	5′-ATTCAAATCCATATCAAAATCTAAC-3′		
AAV3F	5′-ATTTTGATATGGATTTGAATTTGATT-3′	343	23
AAV3R	5′-AAAATATAACACTCATCCACCACCT-3′		
AAV4F	5′-AGGGAGAGAGTTATTTTTATATGTA-3′	372	25
AAV4R	5′-TCTAATTCTCTTTATTCTACTCCTAC-3′		
AAV5F	5′-AAGGTGGTGGATGAGTGTTATATTT-3′	309	15
AAV5R	5′-AACCTAATCCTCCTAAATCCACTACTT-3′		
AAV6F	5′-GGAGAAGTAGTGGATTTAGGAGGAT-3′	298	14
AAV6R	5′-AATTACAAACCCAAACAACCAAATA-3′		
AAV7F	5′-GGAAAGATTATGAGTTTGATTAAAAT-3′	284	15
AAV7R	5′-AAAAAATTCTCATTAATCCAATTTAC-3′		
AAV8F	5′-AATTGGATTAATGAGAATTTTTTTT-3′	315	21
AAV8R	5′-AATAACCTTCCCAAAATCATAATCC-3′		
AAV9F	5′-TGATTTTGGGAAGGTTATTAAGTAG-3′	274	17
AAV9R	5′-ACAAAAAAACAACATCAAATTCATAC-3′		
AAV10F	5′-TGATGTTGTTTTTTTGTAGATAATG-3′	345	10
AAV10R	5′-TAAACCAAATTTAAACTTCCACCAC-3′		
AAV11F	5′-TGGTGGAAGTTTAAATTTGGTTTAT-3′	323	27
AAV11R	5′-AAAAATTCAAAAACCCTCTTTTTC-3′		
AAV12F	5′-AAAAAGAGGGTTTTTGAATTTTTG-3′	152	6
AAV12R	5′-TTCAATCTTTTTCTTACAAACTACTAACC-3′		
AAV13F	5′-TTTGGTTGAGGAATTTGTTAAGA-3′	369	18
AAV13R	5′-TTATAAATAAACAAAACCCAAATTC-3′		
AAV14F	5′-GTTTTTTTTGGTTTGGGAATTAATA-3′	282	12
AAV14R	5′-AAATCTATTAAAATCAAAATACCCCC-3′		
AAV15F	5′-TTGGGTTTTGTTTATTTATAATAATTATTT-3′	217	4
AAV15R	5′-AATATTAAAAAACTTAAAATTAAATCTCTT-3′		
AAV16F	5′-AGATTTATTAATAATAATTGGGGATTT-3′	299	18
AAV16R	5′-TACTCCAAACAATAAAATAAAAAAC-3′		
AAV17F	5′-AGTATGGATATTTTATTTTGAATAA-3′	316	12
AAV17R	5′-AAAAACCAATTCCTAAACTAATCCC-3′		
AAV18F	5′-AGTTAAGGTTTTAGTTTTTTTAGGT-3′	340	12
AAV18R	5′-AAATTAATTATCCTAATTTCCTCTTC-3′		
AAV19F	5′-AATGGTAGAGATTTTTTGGTGAATT-3′	317	9
AAV19R	5′-AACCCCTAAAAATACACATCTCTATC-3′		
AAV20F	5′-AGGTATGGTTTGGTAGGATAGAGAT-3′	340	12
AAV20R	5′-ATCCACAATAAAATCCACATTAACAA-3′		
AAV21F	5′-AGTGGGAGTTGTAGAAGGAAAATAGTA-3′	312	10
AAV21R	5′-TAACCAACTCCATCACTAAAAATTC-3′		
AAV22F	5′-GTTTGTTAATGTGGATTTTATTGTGGAT-3′	360	22
AAV22R	5′-TAACCACTCCCTCTCTACGCGCT-3′		
